# Communicating the molecular basis of cancer cell-by-cell: an interview with Tatsushi Igaki

**DOI:** 10.1242/dmm.024059

**Published:** 2015-12-01

**Affiliations:** Tatsushi Igaki is Professor at the Graduate School of Biostudies, Kyoto University. He is also a Monitoring Editor for Disease Models & Mechanisms

**Keywords:** *Drosophila*, Apoptosis, Cancer

## Abstract

Tatsushi Igaki is currently based at the Kyoto University Graduate School of Biostudies, where he leads a research group dedicated to using *Drosophila* genetics to build a picture of the cell-cell communications underlying the establishment and maintenance of multicellular systems. His work has provided insight into the molecular bases of cell competition in the context of development and tumorigenesis, including the landmark discovery that oncogenic cells communicate with normal cells in the tumor microenvironment to induce tumor progression in a non-autonomous fashion. In this interview, he describes his career path, highlighting the shift in his research focus from the basic principles of apoptosis to clonal evolution in cancer, and also explains why *Drosophila* provides a powerful model system for studying cancer biology.

## Abstract (precis)

**Summary:** We spoke to Tatsushi Igaki about his research using *Drosophila* models to uncover the principles of cell-cell communication in development and cancer. He describes his passion for research and the key events that shaped his career.


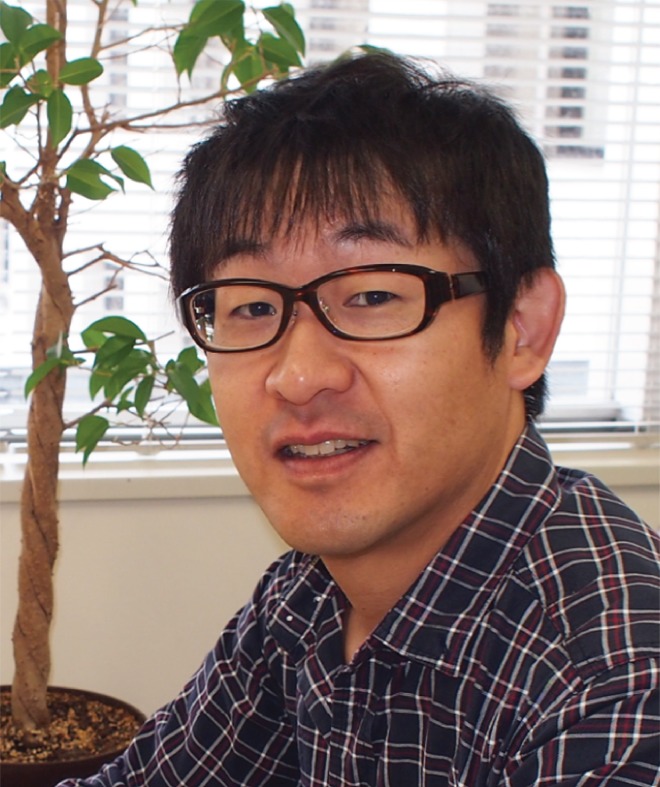


Tatsushi Igaki was born in Okayama, Japan, in 1970. He obtained a Bachelor's degree in Pharmaceutical Sciences from Okayama University, where he carried out research on apoptosis using cultured cells under the supervision of Hikoya Hayatsu and Yusuke Wataya. After graduation, he started working on neuronal apoptosis at the Research Institute of Kyorin Pharmaceutical Company but, after working at the company for 4 years, he went back to academia and enrolled as a graduate student at the Graduate School of Medicine at Osaka University. Tatsushi's first introduction to *Drosophila* as a model came during this time, and he used flies to further explore the molecular underpinnings of apoptosis under the supervision of Masayuki Miura. He then moved on to Yale University to work in Tian Xu's lab, where he harnessed the genetic tools and resources provided by the group to shed light on cell-cell communications and signaling pathways relevant to tumor metastasis. In 2007, he got an independent Assistant Professor position in the Graduate School of Medicine at Kobe University in Japan. He secured tenure a few years later and moved to the Graduate School of Biostudies at Kyoto University as a full Professor. Nowadays, his group uses *Drosophila* as a platform to study cell-cell communication in epithelial tissues, focusing on cell ‘competition’ and ‘cooperation’, with the aim of understanding how multicellular systems are maintained and how dysregulation of these processes leads to cancer.

**Why did you decide to pursue a career in science?**

I first became interested in science in high school, and my interest intensified when I started my first research project as an undergraduate student in Hikoya Hayatsu's lab. In the course of this project I first mastered how to quantify the intracellular dNTP [deoxyribonucleotide triphosphate; dATP, dTTP, dGTP and dCTP] pool using HPLC [high performance liquid chromatography], and my first ‘real’ experiment was to analyze the dNTP pool obtained from mutagen-treated mouse cells, which had been prepared by a senior lab member. The aim of this work was to determine whether the balance in the intracellular dNTP pool was disrupted by mutagen treatment, and my experiment showed that there was no significant effect. However, I realized that there was an additional small peak in my HPLC chromatogram that did not correspond to any dNTP standards. I thought that, because the mutagen used was a nucleoside analog, it has somehow been metabolized within the cell and was utilized for the dNTP pool. I hypothesized that this could be the cause for decreased viability in mutagen-treated cells. Although my colleague did not care about the small peak, I couldn't help thinking about it because I realized that, in the whole world, only I knew about it – this really motivated me to investigate it. Yusuke Wataya, an Associate Professor who directly supervised me, was generous enough to allow me to pursue the finding during my undergraduate work. I really enjoyed the project, and finally found that the small peak was indeed derived from a chemically modified dNTP generated in mutagen-treated cells. It was just a tiny piece of work, like a small peak in my life, but I really learned what science is from the experience – the opportunity to make new and exciting discoveries by following the heart. I decided then and there to spend my life doing research.

“It was just a tiny piece of work, like a small peak in my life, but I really learned what science is from the experience – the opportunity to make new and exciting discoveries by following the heart”

**For your PhD, you investigated mechanisms of apoptosis in *Drosophila*. Were you particularly interested in this topic and in working on *Drosophila* or did you stumble upon the field?**

During my time in industry, I read many papers in the apoptosis field. That period, 1995-1999, was the golden age of apoptosis research. I was attracted by the mechanism of apoptosis, which seemed beautiful in its simplicity. Although I didn't have many opportunities to attend meetings or even discuss advances in the field with other researchers, I followed the field by reading papers. I still clearly remember when I finally decided to go back to academia. It was when I stood reading a new issue of *Cell* [August 21, 1998] in the library during a lunch break. In that issue, I found two back-to-back papers from Junying Yuan's lab and Xiaodong Wang's lab that beautifully demonstrated, using mouse genetics, the molecular basis of why a death-ligand, Fas, causes apoptosis through different pathways in different cell types. They were elegant studies that clearly resolved an important issue, and I was shocked that it had taken a year for the knowledge to get to me, because I wasn't a researcher in the field. I felt that I needed to go back to academia as soon as possible, so I applied for a PhD position in Masayuki Miura's group at Osaka University. During the interview for this position, Masayuki told me that he had just started working on *Drosophila* to understand apoptosis at the organismal level and was going to shift his focus from cultured cells to flies. I had been impressed by fly genetics and I instantly agreed to his direction. Indeed, working with flies has changed my view of science. In contrast to the cultured cells that I had been working with before, first and foremost flies give us insight into the phenomenon of life. We are instantly convinced of the importance of a particular biological process before we start dissecting its molecular mechanism. “Ask living organisms if you want to know the mechanism.” This is one of the most valuable pieces of advice I received from Masayuki Miura, who has always been my idol.

**Can you tell us more about your PhD work? What was your greatest achievement?**

In the course of my PhD, I characterized the first *Drosophila* homolog of Bcl-2 as a prototype of pro-apoptotic Bcl-2 family members, and identified the first and sole ortholog of tumor necrosis factor [TNF]. I named this TNF ortholog ‘Eiger’, after the impressive mountain that I had visited 4 years earlier with my wife. Thirteen years after the discovery, Eiger is now recognized as one of the key molecules that regulate tissue homeostasis and tumor development in flies. The identification of a TNF ortholog implied the existence of mammalian-like ‘context-dependent’ or ‘stochastic’ cell-death regulation, in addition to genetically programmed cell-death mechanisms. Eiger thus gave me the opportunity to dissect complex cell-death regulation through cell-cell communications. I was really interested in this possibility and it made me further recognize the advantages of working in flies – this question couldn't be studied in other invertebrate model organisms such as *C. elegans*, where all cell fates are genetically programmed.

**After your PhD, you moved to Yale University to work in Tian Xu's lab. What stimulated this choice?**

Tian Xu's group had established the FLP/FRT-mediated genetic mosaic technique that enables us to study cell-cell communications in fly tissues by ‘clonal analysis’. I decided to join Tian's lab because, as a continuation of my PhD work, I wanted to use the genetic mosaic technique to study stochastic cell-death regulation through cell-cell communications. Tian's lab had a lot of useful tools and valuable expertise, and I expected that, using their genetic mosaic technique, I would be able to get close to a concept that explains how cell-cell communications underlie the establishment of a complex multicellular system. In addition to this, when I was interviewed by Tian on the phone, he told me about his recently established fly model of tumor metastasis, which they were about to submit to *Science*. This was the first *Drosophila* genetic model of tumor metastasis, in which GFP-labeled tumor cells induced in the larval eye tissues overgrow, degrade basement membrane, migrate out of the eye tissue, and invade into neighboring tissue called ventral nerve cord or metastasize to distant organs. Gene expression can be easily manipulated within tumor cells, and the model can be used to screen for genes involved in metastasis*.* This unique and interesting model, which is now widely used by *Drosophila* labs interested in cancer, further stimulated me to move to Tian's lab.

**Was your experience at Yale important for consolidating your research interests as a PI?**

Definitely. Without my experience in Tian's lab, I would be moving in a different direction now. I spent four and a half years at Yale, and it was definitely one of the most wonderful times for me in both science and life. Tian is a great scientist, with extremely unique ideas and a very logical way of thinking. When I was struggling with my manuscripts, I often drove to Tian's house to have discussions that frequently went on until after midnight. I am sure that all my interactions with him significantly influenced my sense and style of science.

In Tian's lab, I first worked on the signaling mechanisms involved in tumor metastasis, using clonal analysis. During this time, I came across an interesting phenomenon: clones of oncogenic cells deficient for a neoplastic tumor-suppressor gene, *scribble*, are actively eliminated from the epithelial tissue when surrounded by wild-type cells; a process that has now been recognized as a type of ‘cell competition’. This phenomenon was first reported by Helena Richardson's group, but we could finally unveil the underlying mechanism, in which Eiger plays a central role. We found that Eiger is activated in oncogenic *scribble* mutant cells when surrounded by wild-type cells and leads to activation of downstream JNK signaling, which causes elimination of *scribble* mutant cells. It was amazing for me, because the Eiger-deficient flies that I generated as a graduate student gave no phenotype, so I disappointedly thought that Eiger might not be important. I could finally elucidate the physiological role of Eiger, which is latent in the normal situation but is activated when a tissue needs to eliminate dangerous cells such as oncogenic mutant cells: a fail-safe mechanism of the epithelial tissue against neoplastic development. At this point I had a clear vision of what I wanted to work on and knew I was ready to become independent. Since that time I have devoted my research to studying the basic principles of epithelial cell-cell communications in tissue homeostasis, tissue growth and tumorigenesis through clonal analyses.

**Was it, in a way, a natural evolution for you to move from apoptosis to the study of cancer?**

I have been interested in cancer since the early days of my career. As an undergraduate student I was really attracted to molecular biology and felt that cancer would be one of the most interesting questions to be addressing using molecular biology tools. Apoptosis, which is a longstanding interest of mine, is a central aspect of cancer. But I do not think that I have made an active move from basic to more translational research. In my mind, I have consistently focused on the rather fundamental basic principles of biological phenomena, which are indeed often related to cancer. Of course, an ultimate goal is that the concepts emerging from our fly work contribute to development of a revolutionary anti-cancer strategy, but I have consistently approached the question from a basic standpoint. I am now interested in epithelial cell-cell communications, especially cell-cell cooperation and competition, which I believe would lead to the understanding of multicellularity, tissue homeostasis and, ultimately, cancer.

**Your group has contributed to dissecting how certain alterations in oncogenic cells can induce growth and metastatic behavior in the surrounding tissue. Could you tell us more about these findings and the implications for cancer therapy?**

We have found through a genetic screen in *Drosophila* epithelium that clones of cells with oncogenic Ras activation and mitochondrial dysfunction cause their surrounding benign tumors to acquire metastatic abilities – this is termed ‘non-autonomous’ tumor progression. Both Ras activation and mitochondrial dysfunction are frequently observed in human cancers, and we found that, remarkably, these two mutations collaborate to induce drastic tumor progression in neighboring tissue. Importantly, mutant cells with simultaneous Ras activation and mitochondrial dysfunction do not overgrow, but instead they undergo cellular senescence. Thus, such mutant cells behave as ‘oncogenic niche cells’ to constantly promote non-autonomous tumor progression. An implication of our findings from a translational viewpoint is that it reminds researchers to look at cancer as a heterogeneous mutant cell population in which there are constant oncogenic cell-cell communications. Researchers may further find that some other oncogenes or tumor-suppressor genes that we think we understand well also have unexpected, non-autonomous oncogenic activities *in vivo*. This seems to make cancer more complicated but, at the same time, raises hope for cancer therapy because, by delving deeper into cancer biology, we uncover more therapeutic targets. Although we are still far from using this knowledge for cancer treatment, I believe that it will contribute to the development of new cancer therapies in the future. I think the biggest role of *Drosophila* in cancer research is to provide a conceptual advance in understanding the basic biology, which, in turn, informs the drug discovery process.

“An implication of our findings from a translational viewpoint is that it reminds researchers to look at cancer as a heterogeneous mutant cell population in which there are constant oncogenic cell-cell communications”

**What makes *Drosophila* a good model for understanding cancer biology?**

The biggest advantage to using *Drosophila* for studying tumorigenesis, at least for me, is the ability to apply clonal analysis in the model. Tumors arise from a single or a few mutant cells emerging from a normal epithelial sheet. Such mutant cells undergo clonal expansion and evolution into cancer cells via cell-cell communication between mutant cells and surrounding normal cells. *Drosophila* is the only genetically tractable model animal that we can use to systematically study such oncogenic cell-cell communication at the organismal level. This is enabling us to gradually dissect the basic principle of clonal evolution in the context of cancer. It is also a crucial benefit for my work that, in *Drosophila*, we can perform unbiased genetic screens that provide us with clues for solving the many complex mysteries of biological systems, which are otherwise often impenetrable. Of course, *Drosophila* has some obvious limitations for studying tumor growth and metastasis, as it lacks a vascular system, the adaptive immune system, typical epithelial-mesenchymal cell interactions and postnatal cancer. But I think these are small issues and can be overcome. Flies have many other advantages, and I am overall very pleased to work with this beautiful animal with two wings and six legs.

**What recommendations would you give to PhD students and young researchers striving to successfully pursue a career in science?**

I don't consider myself to be in the position to be able to give advice to young scientists, but one thing I would say is that it was crucial for me to work out my lifetime interest as a PhD student and postdoc. Any kind of science is interesting, but what we can do in the course of our careers is very limited. I hoped to eventually become a PI and be able to bet my life on my own science, so, I knew that the question I chose should be unique, enigmatic and, most importantly, fun to tackle. Trying to pin down my lifetime interest made me think more about science in general. I think it is important to be confident in your choices of research interest, whatever other people think. In my case, I knew that I wanted to dedicate my career to cell-cell communication. I felt that there was a huge gap between what we know about the molecular biology of the cell and what we know about how the multicellular system works. It was so intriguing for me to think about how cells communicate, cooperate and compete with each other to develop a complex multicellular system, and to think about pathologies such as cancer as a failure of normal cell-cell communication. I also tell my students in the lab that scientists are just like athletes. Our scientific knowledge, sense, originality and creativity actually come from everyday hard work, so it is very important for us to keep up our daily training – just like athletes. These efforts are rewarded in science, making this career path a worthwhile investment of life.

“Our scientific knowledge, sense, originality and creativity actually come from everyday hard work, so it is very important for us to keep up our daily training – just like athletes”

**What would you like to achieve in the next decade?**

I would like to fully understand the basic principles of how cells develop and maintain a multicellular network. Towards this goal, my lab is continuing its efforts to understand the molecular basis of cell-cell cooperation and competition in the epithelial tissue. We are currently performing more than 15 genetic screens in *Drosophila*, which I believe will provide us with some answers in the next decade, and these could also contribute to the development of new therapeutic strategies against cancer.

**Moving on from your research, what do you enjoy doing outside the lab?**

I love watching movies and doing sports, especially baseball and running marathons, as well as climbing mountains with my wife.

